# Combinatorial Expression Rules of Ion Channel Genes in Juvenile Rat (*Rattus norvegicus*) Neocortical Neurons

**DOI:** 10.1371/journal.pone.0034786

**Published:** 2012-04-11

**Authors:** Georges Khazen, Sean L. Hill, Felix Schürmann, Henry Markram

**Affiliations:** Blue Brain Project, Brain Mind Institute, Ecole Polytechnique Fédérale de Lausanne, Lausanne, Switzerland; Mount Sinai School of Medicine, United States of America

## Abstract

The electrical diversity of neurons arises from the expression of different combinations of ion channels. The gene expression rules governing these combinations are not known. We examined the expression of twenty-six ion channel genes in a broad range of single neocortical neuron cell types. Using expression data from a subset of twenty-six ion channel genes in ten different neocortical neuronal types, classified according to their electrophysiological properties, morphologies and anatomical positions, we first developed an incremental Support Vector Machine (iSVM) model that prioritizes the predictive value of single and combinations of genes for the rest of the expression pattern. With this approach we could predict the expression patterns for the ten neuronal types with an average 10-fold cross validation accuracy of 87% and for a further fourteen neuronal types not used in building the model, with an average accuracy of 75%. The expression of the genes for *HCN4*, *Kv2.2*, *Kv3.2* and *Caβ3* were found to be particularly strong predictors of ion channel gene combinations, while expression of the *Kv1.4* and *Kv3.3* genes has no predictive value. Using a logic gate analysis, we then extracted a spectrum of observed combinatorial gene expression rules of twenty ion channels in different neocortical neurons. We also show that when applied to a completely random and independent data, the model could not extract any rules and that it is only possible to extract them if the data has consistent expression patterns. This novel strategy can be used for predictive reverse engineering combinatorial expression rules from single-cell data and could help identify candidate transcription regulatory processes.

## Introduction

Experimental and computational informatics studies have revealed more than 270 genes associated with voltage-gated ion channels in the *Rattus norvegicus* (Gene Ontology: GO:0005244 as of January 2011). It is the combinations in which these genes are expressed as well as the precise spatial distribution and biophysical properties of the channels they code for that underlies the diversity of neuronal electrical properties [1].

Previous studies have localized and identified the distribution of ion channels in specific neurons [2], [3], [4], [5], [6] and attempted to match gene expression profiles with different neuronal cell types based on their electrical or morphological characteristics [6], [7], [8], [9], [10], [11]. Although these studies provided important insight into the correlation between single ion channels and the electrical behavior of neurons, they do not address combinatorial rules of gene expression in different classes of neurons. A more comprehensive strategy would be to identify preferred combinations of expressed genes in morphologically and electrically diverse neurons located in different regions.

The most extensive multivariate study was carried out by Toledo-Rodriguez *et al*. [12], where the patch-clamp technique (see *Materials and Methods*) was used to characterize the electrical and morphological properties from 203 juvenile rat neocortical neurons from layers 2 to 6, while simultaneously performing single-cell multiplex RT-PCR for twenty-six ion channel genes on the aspirated cell cytoplasm. We used the same dataset from Toledo Rodriguez *et al.* and asked whether combinatorial rules could be extracted. It is worth noting, that the neurons in the Toledo Rodriguez *et al.* study were selected based on the expression of the house-keeping gene *Gapdh* and that only those expressing this gene and a minimum of two ion channel genes were used in the analysis resulting in only 203 out of the 601 initially harvested. The abbreviations used for the morphological and electrical phenotypes were previously defined in [13], [14] (Table 1). The major advance of the study by Toledo Rodriguez *et al.* was that it enabled the identification of clusters of ion channel gene expression, correlations between genes expressed and the electrical phenotypes, and the further prediction of electrical properties of neurons from expression profiles.

**Table 1:** Different layers, morphological and electrical phenotypic profiles of the 65 neurons.

Layer (L)	Morphological Identity (M)	Electrical Identity (E)
L2/3	Large Basket Cell (LBC)	Continuous Adapting (cAD)
L4	Martinotti Cell (MC)	Continuous Fast Spiking (cFS)
L5	Nest Basket Cell (NBC)	Delayed Fast Spiking dFS
L6	Pyramidal Cell (PC)	Continuous Stuttering (cST)

**Figure 1:**
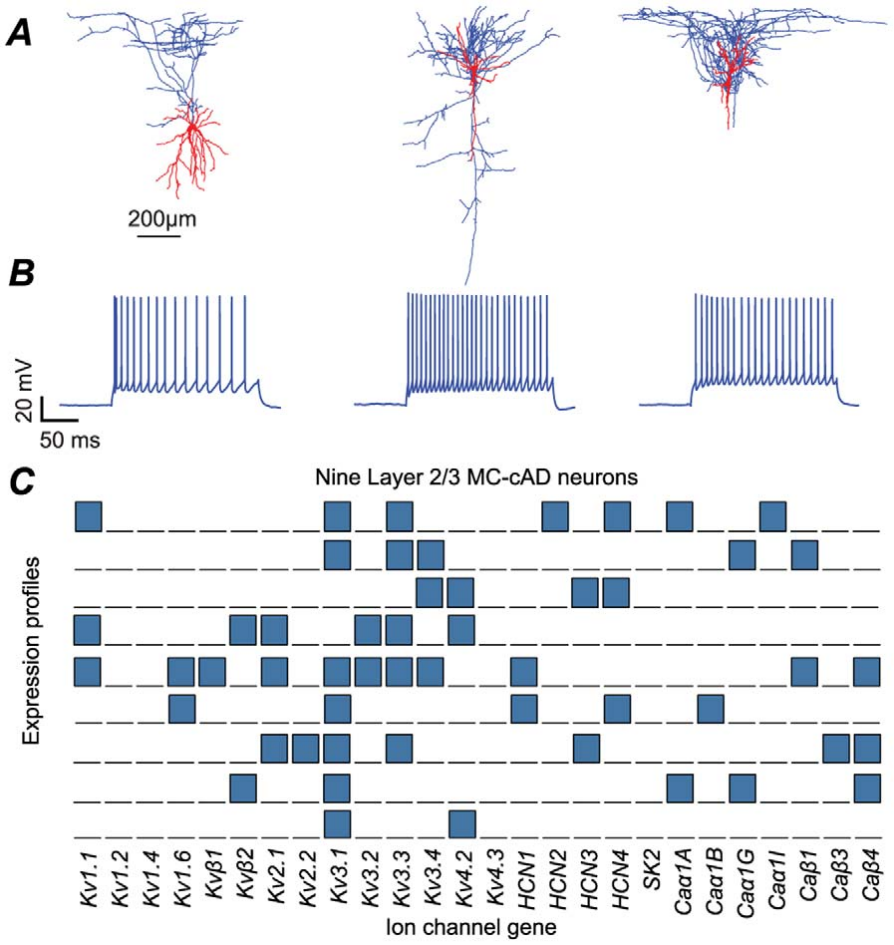
Diversity within layer L2/3 MC-cAD neurons. ***A*** Reconstructed morphologies of three L2/3 MC-cAD neurons. ***B*** Electrical response of the same three L2/3 MC-cAD neurons. ***C*** Genetic profiles of the twenty-six ion channel genes in the nine L2/3 MC-cAD neurons.

By exploring this data, however it also becomes clear that expression profiles are distinctive not only between neurons displaying different electrical behaviors and morphologies, but also within neurons even of the same morpho-electrically classified neuron type located in the same neocortical layer. For example, the variability of the expression profiles in the classical adapting Martinotti Cell (MC-cAD) found in layer L2/3 neurons is clearly noticeable (Figure 1). Is this finding just due to experimental artifacts or does such variability reflect combinatorial expression rules? Standard statistical correlation and multivariate analyses are not sufficient to reveal the preferred combinations of expressed genes in different classes of neuron. Even for the twenty-six ion channels we examined, there are over 67 million theoretically possible combinations (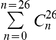), let alone the actual number of possible combinations for the entire channelome of a cell.

Our approach to this problem was to build a combinatorial rule extractor model that can be constrained by, in principle, any information about the neurons that may be available. We classified neurons according to their morphological (M), electrical types (E) and the neocortical layer (L) in which they resided. Even though it is still not possible to objectively classify neuron types, we reasoned that there would be sufficient information in this generally accepted subjective classification to at least partly constrain the model. We constructed the combinatorial rule extractor model as multiple incremental Support Vector Machines (iSVM), one for each of the twenty-six ion channel genes where we searched for the best combinations of input genes that would improve the prediction of the expression of each other gene. By exploring these preferred combinations we were able to derive candidate expression rules governing each gene and then also the preferred combinations expressed in each neuronal type. This combinatorics showed that the same type of neuron can indeed express different combinations of ion channel genes. We constructed these models using ten neuronal types, each having more than three neuron counts, out of the twenty-four in our dataset. Additionally, in order to assess the significance of the models and the extracted rules, we fitted the iSVM models on a completely random dataset where the expression value for each gene was sampled randomly with a Bernoulli distribution having the probability of expression equal to the experimentally determined expression frequency. We show that this approach can effectively reveal combinatorial expression rules from the experimental data but could not extract any rule from the random data and that the observed gene expression variability can be explained in part by these rules and is not random. This approach revealed a spectrum of novel candidate gene expression combinations that provide new insight into the regulatory mechanisms of ion channel gene expression.

## Results

The data pre-processing step resulted in one hundred and thirty-five neurons. The models were built using sixty-five neurons grouped into ten different combinations of layer (L), morphology (M), and electrical type (E) (LME) each of which having a neuron count greater than 3 (Table 2). The data used to build the model is referred to as *model data* hereafter. The remaining data consisted of seventy neurons of which only eighteen were retained because their L, M and E parameters were found in the *model data* but their LME combinations were not (*see* *Materials and Methods*). These eighteen neurons belonged to fourteen different LME combinations (Table 3) each of which having only one or two neuron counts and are referred to as generalization data hereafter (*see* *Materials and Methods*).

**Table 2:** Layer, morphology and electrical type combinations (LME) of the model dataset with the corresponding neurons counts.

LME Type	Neuron Count
L2/3 LBC-cAD	7
L2/3 LBC-cFS	7
L2/3 LBC-dFS	4
L2/3 MC-cAD	9
L2/3 NBC-cFS	12
L4 LBC-cST	4
L4 MC-cAD	6
L5 MC-cAD	5
L5 PC-cAD	5
L6 PC-cAD	6

**Table 3:** Layer, morphology and electrical type combinations (LME) of the generalization dataset with the corresponding neurons counts.

LME Type	Neuron Count
L2/3 PC-cAD	2
L2/3 NBC-cAD	1
L2/3 LBC-cST	1
L2/3 NBC-dFS	1
L4 PC-cAD	1
L4 LBC-dFS	1
L4 LBC-cFS	2
L4 NBC-cAD	1
L4 NBC-dFS	1
L5 NBC-cFS	2
L5 LBC-dFS	1
L5 LBC-cFS	2
L5 MC-cFS	1
L6 LBC-cST	1

### Correlative expression

**Figure 2:**
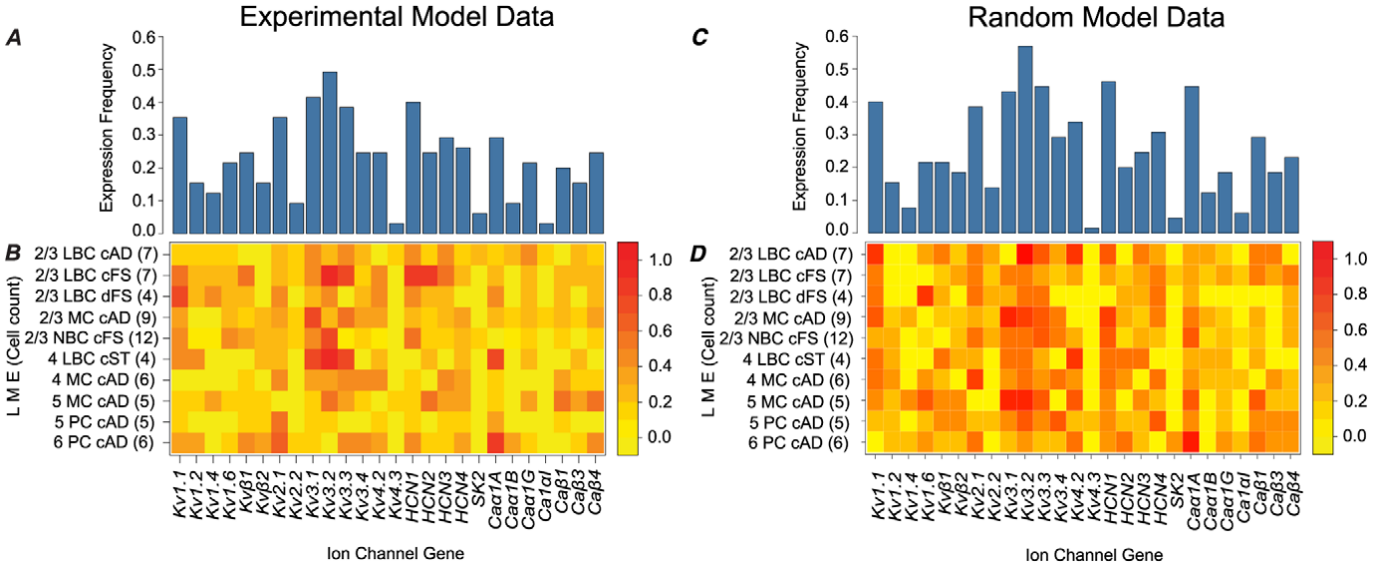
Gene expression frequencies of the twenty-six ion channel genes in the model dataset. ***A*** Overall expression frequency of the twenty-six ion channel genes in the 65 neurons of the experimental model dataset. ***B*** Expression frequencies of the twenty-six ion channel genes in the ten neuronal types of the experimental model dataset. ***C*** Overall expression frequency of the twenty-six ion channel genes in the 65 neurons of the random model dataset. ***D*** Expression frequencies of the twenty-six ion channel genes in the ten neuronal types of the random model dataset.

No single ion channel gene is ubiquitously expressed and the highest individual frequency of expression was for *Kv3.2* (∼50% of cells). Each of the twenty-six genes had an overall expression frequency less than 50% in the model data set (Figure 2A). The observed average expression frequency for all ion channel genes is 23.07%. The potassium channel genes coding for *Kv1.1*, *Kv2.1*, *Kv3.1*, *Kv3.2*, *Kv3.3*, and the cationic channel gene *HCN1* have the highest frequency of expression (greater than 35%) while the *Kv2.2*, *Kv4.3*, *SK2* and *Caα1I* channel genes have the lowest frequency of expression (less than 10%) (Figure 2A). It is expected that neurons only express a small fraction of the ion channels [13], [15], but the low expression frequency of their genes might also be due to the drawback of single-cell gene expression profiling and specifically multiplex RT-PCR where a significant amount of false negatives may be present because of mRNA harvesting or amplification failures [12], [16].

Some of the genes tend to have a relatively similar level of expression between LME types hence it is likely that there is no significant relationship between the LME type and the expression of these genes (Figure 2B). However, many of the channel genes appear to be differentially expressed in different LME types. For instance, *Kv3.3* tends to be highly expressed in L2/3 LBC-cFS and L4 LBC-cST neurons, while very lowly expressed in L2/3 LBC-dFS, L2/3 NBC-cFS and L5 PC-cAD neurons. *HCN1* and *HCN2* seem to be expressed the most in L2/3 LBC-cFS. *Caα1A* is highly expressed in L4 LBC-cST and L6 PC-cAD as opposed to L2/3 NBC-cFS neurons where its expression is less than 10%. *Kv1.2* and *Kv1.4* are almost absent in L2/3 MC-cAD, L2/3 NBC-cFS, and L5 PC-cAD neurons while expressed in almost 60% of L2/3 LBC-dFS neurons. The expression of *SK2*, *Caα1B*, and *Caα1I* channel genes were not detected in layers 4, 5, and 6 neurons. Although these results cannot be generalized because of the small sample size (n = 65) they indicate that some relationship between the expression frequencies and LME type of neuron exists and suggest combinatorial expression rules may also exist.

### Model selection

In order to extract combinatorial rules, we first needed to select a model capable of assessing and prioritizing the value of single and combined genes for the prediction of gene expression –i.e. how well do expressed genes predict expression of other genes. To find the optimal model, we began by comparing the performance of two well-established classifiers, Logistic Regression (LR) [17] and Support Vector Machines (SVM) [18]. Both classifiers were initially fitted for every ion channel gene using the three categorical variables: Layer, Morphology, and Electrical type as input parameters.

The performance of SVM classifiers depends on two main parameters, the cost parameter *C* and the kernel parameter *γ*, and on the kernel function used. The cost parameter *C* regulates the tradeoff between allowing training errors and forcing rigid margins while the *γ* parameter determines the width of the Radial Basis Function (RBF). We performed an extensive grid search, 961 grid points per model, and used a 10-fold cross validation to find the best *γ* and *C* parameters for every ion channel using both linear and radial kernels. The final values of these parameters yield a model for predicting the expression of a single ion channel gene. We found that the accuracy of the models with radial kernel is marginally better than that of the linear ones (difference <1%) and that the best twenty-six *γ* and *C* parameters fall within the ranges [3.05e-5, 2] and [0.03125, 32] respectively (Figure S1).

**Figure 3:**
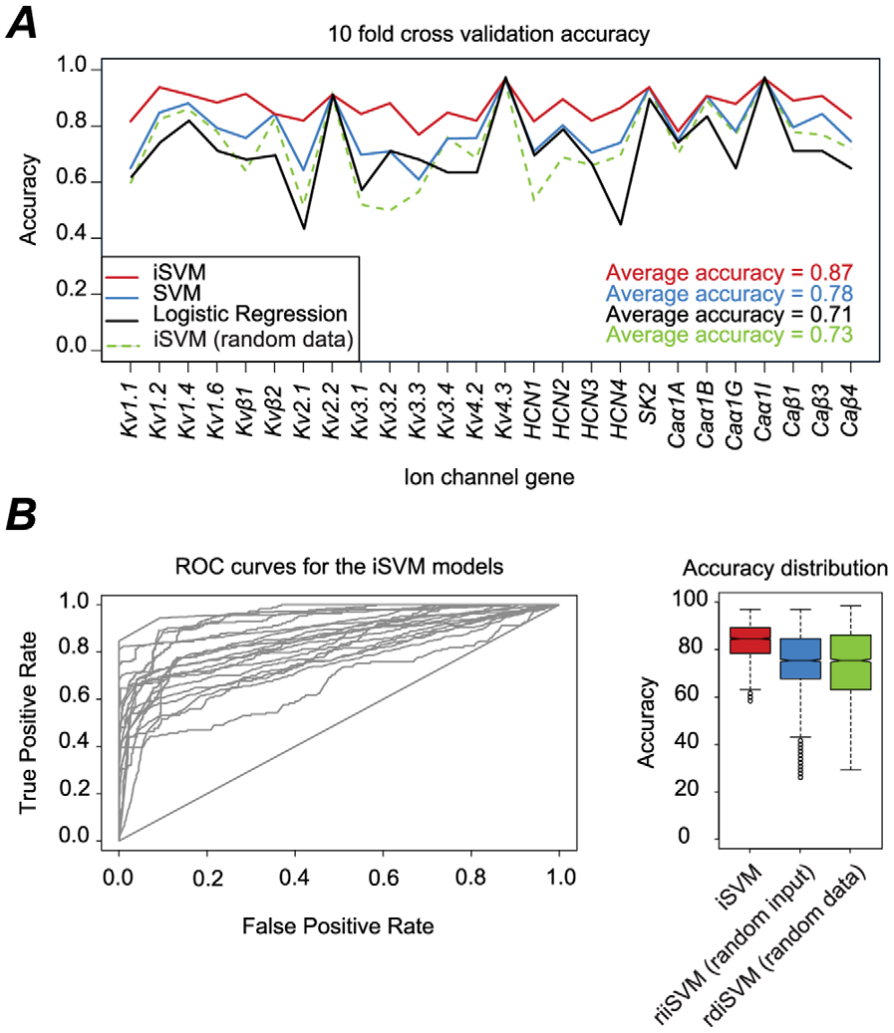
Models assessment and accuracy. ***A*** 10-fold cross validation accuracies of the Logistic Regression (black), base SVM models (blue), and the iSVM models (red) of the twenty-six ion channel genes. ***B***, left, Receiver Operating Characteristic (ROC) curves of the iSVM models for the twenty-six ion channel genes. ***B***, right, boxplots of the 10-fold cross validation accuracies of the iSVM model (red), random inputs iSVM (riiSVM) (blue), and random data iSVM (rdiSVM) (green) after 1000 iterations.

We estimated the accuracy of the LR and SVM classifiers by performing a 10-fold cross validation on the *model data* set (see *Materials and Methods*). For most ion channel genes, the tuned SVM models out-performed the LR ones and the overall 10-fold cross validation average accuracy of the SVM model was found to be 78% as opposed to 71% for the LR Model (Figure 3*A*). Based on these results, the SVM model was selected as the base model with the three input parameters, L, M and E.

In order to capture combinatorial expression rules of all ion channel genes that best fit with the expression of any one gene (the *i*th ion channel gene), we developed an incremental SVM classifier (iSVM) by iteratively combining the expression profiles of the remaining genes to the LME input parameters of the SVM model and evaluated the improvement in the prediction. At the first iteration, we identified which gene out of the twenty-five remaining genes improves the prediction accuracy of the *i*th gene the most and retained it as an additional input parameter in the model (input gene 1, IG1). We then repeated the same process in the second iteration by sequentially combining each of the remaining twenty-four genes in turn to the IG1-LME model and identified which gene of the twenty-four improved the prediction accuracy the most and then retained it as well (IG2) for the third iteration. We iterated until the prediction accuracy of the *i*th ion channel gene could no longer be improved.

An iSVM model was generated for each of the twenty-six ion channel genes. Two of the iSVM models, for ion channels *Kv1.2* and *Kv3.3*, had a high cost parameter C = 32 while most of the remaining ones had a low cost parameter value (C<5). A high cost parameter increases the penalty for non-separable points which can create a more accurate model at the risk of having some over-fitting [19]. On the other hand, the models with the lowest cost parameter (around C = 0.03) are the ones where the channels have the lowest expression frequency (<10%) indicating a possible under-fitting. In fact, the prediction of the expression of these channels was always 0 (*Kvβ2*, *Kv2.2*, *Kv4.3*, *SK2*, *Caα1B*, and *Caα1I*).

The maximum number of input genes needed to reach the peak level of prediction for any gene was found to be 5 (Figure S2). Using more than 5 input genes resulted in reduced accuracy for some genes and no improvement for others. It is interesting to note that although correlation coefficients have proven beneficial to reconstruct biological networks [20], [21], [22], they were not found to be useful in our analysis because of their low values (mean, 0.038±0.146) and were not used as a criterion for choosing the input genes. The highest absolute correlation coefficient is 0.48 (Figure S3). Some ion channel genes have a high correlation coefficient in one neuronal type and low in another. If our selection were to be based on correlation, we would then need to split the data based on the neuronal types and then compute the correlation coefficients for every neuronal type. However, this might only work for identifying linear relationships between the input and target genes, which might not necessarily be the case. Thus, we found it more appropriate to base our selection criterion on the actual improvement in the accuracy of the prediction.

The prediction accuracy increased for all genes with the exception of the six genes with very low expression frequency, *Kvβ2*, *Kv2.2*, *Kv4.3*, *SK2*, *Caα1B*, and *Caα1I* (Figure 3A). In fact, the prediction of these genes is trivial and is always estimated to be 0 irrespective of the model used which explains the fact that the incremental steps did not improve their prediction. The accuracy of the iSVM was increased beyond that of the SVM in some cases by more than 17% (*Kv1.1*, *Kv3.1*, *Kv3.3*) and the overall average accuracy was significantly improved from 78% for the SVM model to 87% for the iSVM model (*P* = 3.78e-06) when taking all genes, and from 74.8% for the SVM model to 85.6% for the iSVM when excluding the six genes with the trivial 0 prediction. This demonstrates that specific combinations of ion channel genes are preferred in specific LME types.

The iSVM models can identify which ion channel genes are sensitive to other combinations of expressed genes and can highlight the neuronal type where these particular preferred combinations occur. However, these models cannot specify the *type* of relationship within these combinations such as: an AND relation (both expressed or both not expressed), a NOT relation (one expressed while the second not expressed), and an OR relation (any one of the input genes is expressed). Given that the expressions are binary, one can apply Boolean minimization functions such as ESPRESSO [23] to extract the types of relationships between the genes. While a logic gate model can easily be constructed to derive the expression rules [23], because of the small sample size in this study we could directly extract the types of relationships by simply noting the AND, NOT and OR relationships between the genes (Tables S1, S2).

### Model assessment

In order to assess the significance of the iSVM for every ion channel gene, we computed the accuracy of the iSVM models on a completely random dataset where the expression value for each gene was sampled randomly with a Bernoulli distribution having a probability of expression equal to the observed experimental expression frequency. The random data was divided into model and generalization dataset with the same neuron types and counts as the experimental data (Figure 2E, F, G, H) (referred to as iSVM random data, rdiSVM) (see *Materials and Methods*). The rdiSVM models have an average 10-fold cross validation accuracy of 73% on the random data. None of the models outperformed the normal SVM models (Figure 3A) and no improvement was detected after incrementing the number of inputs (Figure S1) (the models for the five genes with the lowest frequency of expression (<10%) had the same accuracy). Consequently, none of the input genes improved the prediction of others and no combinatorial expression rules could be extracted from the random data. This indicated that our extractor model could not identify any combinatorial expression rules in the random data.

In order to assess the uniqueness of the iSVM models we also randomly varied the iSVM input genes, as well as the gamma *γ* and cost *C* parameters (referred to as iSVM random inputs, riiSVM) and then recomputed the accuracy. Figure 3B shows the boxplots of the accuracy distributions of the iSVM (red), riiSVM (blue) and rdiSVM (green) after 1000 iterations. The analysis of variance (ANOVA) of the accuracies of the three models showed that there is a significant difference between them at the 5% level (*P*<2.2e-16). Additionally, when specifically comparing iSVM to riiSVM and iSVM to rdiSVM we also found that there is significant difference at the 5% level (*P* values<2.2e-16). These results clearly indicate that the fit of the iSVM models, for the ten neuron types, is significant at the 5% level and that the combinations of expressed ion channels cannot be obtained randomly in these neuron types. There is also a clear relationship between the target ion channel gene to be predicted and the selected input genes as well as the *γ* and *C* parameters, and by only choosing the right set of input genes we can achieve this high level of accuracy.

Additionally, the average Area Under the Curve (AUC) of the twenty-six iSVM models is 77% (Figure 3B). The classifiers having an AUC = 50% (diagonal line) correspond to the five ion channel genes that have the lowest expression frequency (<10%). Although the models for these five ion channels have the highest prediction accuracies (>90%), their AUC is the lowest since they cannot discriminate true positives from true negative and always predict the expression to be 0. The average AUC for the remaining classifiers is 85.4% indicating that the iSVM classifiers can identify true positives in more than 85% of the cases.

### Network diagram

**Figure 4:**
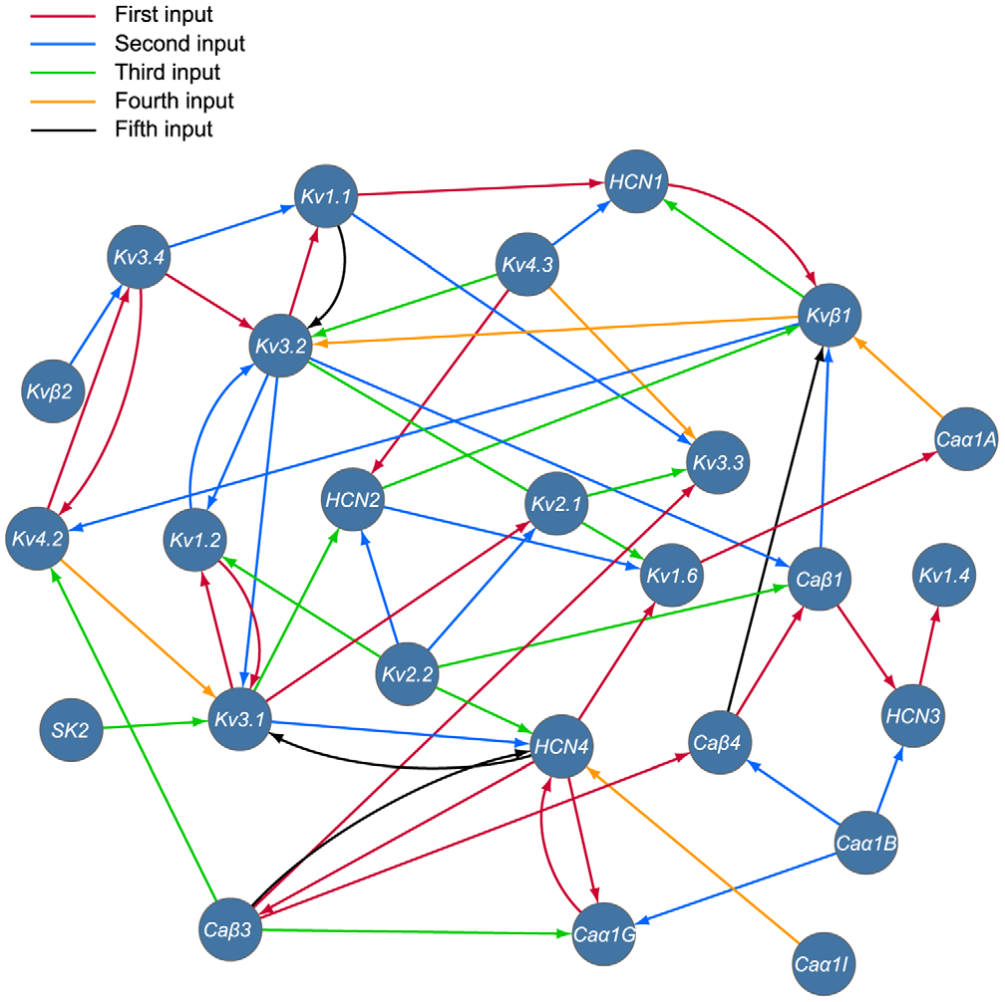
Directed network diagram that links the input ion channel genes to their corresponding output channels. The edges are colored in red, blue, green, orange, and black based on the predictive value of the input ion channel gene. Red edges have the highest predictive value and represent the genes that were selected as inputs at the first incremental step while the black edges have the lowest predictive value and represent the genes that were selected at the last incremental step.

**Figure 5:**
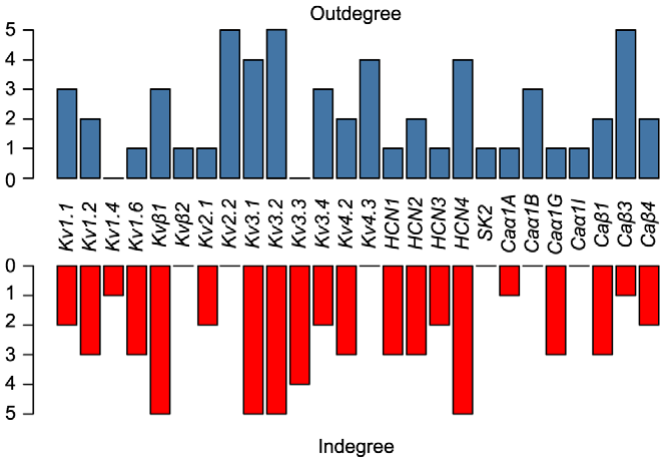
Indegree and outdegree of the twenty-six ion channel genes. The indegree represents the number of input channels used in the iSVM model of a given channel, and the outdegree, number of times a given channel was used as an input for another channel.

The outcome of the iSVM models allows the reconstruction of a directed network diagram that indicates for each gene whether it has predictive value for any of the other genes. The link indicates the direction but not the type of relationship (ie. AND, NOT, OR) (Figure 4) and its color indicates the sequence of the iteration in which the gene was selected. This also highlights the level of improvement in predicting the target gene where the first have the highest prediction value. The channels that have the highest predictive value are *HCN4, Kv3.1 and Caβ3. HCN4* was selected at the first incremental step for three channels (*Kv1.6*, *Caβ3, and Caα1G*) *while Kv3.1* and *Caβ3* were selected at the first incremental step for two channels. *Kv3.1* was selected for *Kv1.2* and *Kv2.1*, and *Caβ3* was selected for *Caβ4* and *Kv3.3*. Additionally, channels *Kv2.2*, *Kv3.2* and *Caβ3* have the highest outdegree (see *Materials and Methods*) and were used as input parameters for five different channels, while *Kv1.4* and *Kv3.3* were not found to be useful for predicting any channel (Figure 5). Channels *Kv3.1*, *Kv3.2*, *HCN4*, and *Kvβ1* have the highest indegree and each have five input channels in their iSVM model (Figure 5). The network diagram (Figure 4) indicates that *HCN3* has a predictive value for *Kv1.4* and is the only input channel for it. The prediction accuracy of *Kv1.4* improved by 4% after the inclusion of the expression of *HCN3* in its iSVM model (87% for the base SVM model and 91% for the iSVM, Figure 3A).

Since the iSVM model additionally incorporates the input genes as predictors, we first examined the neuronal types where the iSVM model outperformed the SVM model and then extracted a candidate expression rule between the input and the target ion channel genes. We then explored the expression of the input and target genes in these neuronal types for any consistent AND, NOT, or OR relationship. These rules were derived from the model dataset that contains four or more of each of the ten types of neurons. Using this approach we extracted a spectrum of observed rules that explain the combinatorial expression of twenty ion channels (Table S1). No rules could be identified for the remaining six genes (*Kvβ2*, *Kv2.2*, *Kv4.3*, *SK2*, *Caα1B*, and *Caα1I*) because of their low expression frequencies and none of the rules was ubiquitously observed in all of the ten neuron types.

We found for example, that *Kv1.4* is expressed in L5 MC-cAD and L2/3 LBC-dFS neurons whenever *HCN3* is expressed, and is not expressed if *HCN3* is not expressed (Table S3). The base SVM model predicted a 0 value for *Kv1.4* for those neuronal types while iSVM predicted 1 whenever *HCN3* was included as “expressed” and 0 whenever it was included as “not expressed”. Another relationship that the iSVM model was able to identify is between the *Caα1A* and *Kv1.6* channels (Table S4). These two channels have exactly the opposite expression profile in the L6 PC-cAD neurons –i.e. *Caα1A* is expressed only if *Kv1.6* is not expressed and vice versa. All identified expression patterns of the ion channel genes are listed in (Table S1). Rules that were found in more than one neuron type are highlighted in bold and the number of times each rule is observed in the respective neuron types is reported in Table S1. For instance, the *Kv1.4 = HCN3* rule was found in eight out of the nine L5 MC-cAD and L2/3 LBC-dFS neurons (Table S3). *Kv1.4* is expressed in the 3 cases where *HCN3* is expressed, it is not expressed in the 5 cases where *HCN3* is not expressed but was expressed only once when *HCN3* was not expressed. Consequently, the number of occurrence of the *Kv1.4 = HCN3* rule is 8 out of 9. All rules were found in more than 80% of the cases in their respective neuron types with the exception of the *HCN1* rule (*HCN1 = Kvβ1* OR *Kv1.1* in L2/3 NBC-cFS and L2/3 LBC-cFS neurons) and *Kv3.3* rule (Kv3.3 = Kv4.3 OR Kv2.2 OR Kv1.1 OR Caβ3 in L2/3 LBC-cAD neurons) which were found in only 13/19 (68%) and 4/7 (57%) cases respectively.

Our results show that the iSVM model can reveal which ion channel genes may have rules that relate their expression pattern within a specific neuronal type and we therefore extracted and grouped these expression rules for the ten neuronal types of the model data set (Table S2). Interestingly, we found that the majority of the expression rules are consistent whenever identified in different neuronal types, which indicates that these rules are not specific to a single neuronal type. For example, the expression of *HCN3* is similar to that of *Caβ1* in L2/3 LBC-dFS, L4 MC-cAD, and L5 MC-cAD neuronal types (Table S1, S2). Additionally, *Kv1.2* is expressed in L2/3 NBC-cFS, L2/3 LBC-cAD whenever *Kv2.2*, *Kv3.1*, and *Kv3.2* were simultaneously expressed (*Kv1.2* = *Kv2.2* AND *Kv3.1* AND *Kv3.2*), and it is expressed in L2/3 LBC-cFS, L2/3 LBC-dF, L4 LBC-cST, and L5 MC-cAD neuronal types whenever *Kv3.1* and *Kv3.2* are expressed (*Kv1.2* = *Kv3.1* AND *Kv3.2*) (Table S1, S2). The expression rule for *Kv1.6* in L2/3 LBC-cAD and L2/3 NBC-cFS types was also found to be the same (*Kv1.6* = *Kv3.2* AND (NOT (*HCN2*) AND NOT (*HCN4*))) (Table S1). The expression of *HCN1* was consistently related to that of *Kvβ1* in L6 PC-cAD, L5 MC-cAD, 5PC-cAD, L2/3 NBC-cFS and L2/3 LBC-cFS neurons. We did find few cases where a rule did not apply across different cell types, such as the expression rules for *HCN2*. The *HCN2* expression rule in L2/3 LBC-cAD neurons is *HCN2* = *Kv3.1* AND *Kv2.2* AND NOT *Kv4.3* while in L5 MC-cAD it is *HCN2* = NOT *Kv3.1* AND NOT *Kv2.2* AND NOT *Kv4.3* (Table S2). Given the small sample size, it is however not clear if this difference is due to the neuronal type, to other unobserved parameters to an uncertainty introduced by the low neuronal count for this example or to the high false negative rate.

Additionally, we used the in situ hybridization slices of the P14 developing mouse brain from the Allen Brain Atlas (http://developingmouse.brain-map.org/) to check whether some of the identified rules can be found at a lower resolution. In fact, the genes *HCN1* and *Kvβ1* were found to have relatively similar expression patterns in the somatosensory neocortical area, which is consistent with the predictions of our model (Figure S4). The genes *Kv1.2*, *Kv2.2*, *Kv3.2* were also found to have relatively similar expression patterns, which is partly in line with our extracted rule (*Kv1.2* = *Kv2.2* AND *Kv3.1* AND *Kv3.2*) (Figure S5). The expression patterns of the genes *Kv3.1*, *HCN3*, *Kv1.6 and Caβ3* could not however be checked because they were not found in the P14 developing brain.

Given that none of rdiSVM (iSVM with random data) models improved the prediction accuracies of the ion channel genes, no rules could be extracted from these models and no relationship could be identified between the input and target genes of the rdiSVM models. This clearly indicates that our models can only extract rules whenever they are consistently observed in the data.

### Testing the generalization of the models

**Figure 6:**
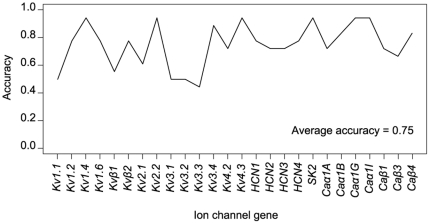
Generalization dataset accuracy for iSVM models of the twenty-six ion channel genes. The generalization data set consists of eighteen neurons belonging to fourteen LME combinations.

We checked to see whether these rules apply to other neuron types having different combinations of Layer, Morphology and Electrical type parameters that were not used in the training set. This would be very beneficial because it is neither efficient nor feasible to identify every single combination of the parameters in brain slices and then perform single cell RT-PCR on each type. We therefore used the iSVM models to predict the expression of the twenty-six ion channels of new neuronal types in the generalization data set. Only new combinations of already observed Layer, Morphology, and Electrical type parameters were used. Figure 6 shows the average accuracy per LME type in the generalization data set, which consists of 18 neurons belonging to 14 neuron types. The average accuracy of the generalization data set was found to be 75%. Given the small difference in performance between the test and training data results for twenty ion channels, it is highly probable that there is no over-fitting and these models can indeed be used to estimate the expression of these twenty ion channel genes. Nonetheless, it is worth mentioning that this drop in accuracy could also be due to the high false negative rate (66%) and that most of the LME neuronal types in the generalization data set used have only one occurrence, so additional data is obviously needed in order to provide even stronger assessment of the generalizability of the models.

## Discussion

We present a computational multi-parametric approach for extracting combinatorial expression rules of ion channel genes in ten different neuronal types of the neocortex. Using the iSVM approach, we developed a strategy to explore the diverse combinations of expressions when constrained by different input parameters, such as the electrical and morphological phenotypic profile, the location of a neuron in a microcircuit as well as the expression of other ion channel genes. We constructed a network diagram that illustrates the predictive value of the expression of single and combined gene with respect to one another (Figure 4) and carried out a logic gate analysis that suggests a spectrum of preferred combinations of ion channels in ten different neuron types.

Ten fold cross-validation was used to estimate the prediction accuracy and significance, and the model was tuned to find the best hyper-parameters *C* and *γ* (see *Results*). In addition to improving the prediction accuracies, the iSVM models identified which combinations of ion channel genes are preferred in different neuronal types. The major advantage of the iSVM model approach is therefore that it allows the identification of different patterns of expressions for the same set of ion channels in different neuronal types. Interestingly, many of the preferred combinations found in any specific neuron type, were then also found in other neuronal types, suggesting that the expression of preferred combinations of ion channels are not necessarily unique to a single neuron type. Additionally, since no rules were extracted from the random data but only the ones with high occurrence frequency (>80%) were identified in the experimental data, we can be confident about the robustness of the extractor model and the significance of the rules in the ten neuron types. Further study would be required to determine whether these preferred combinations we identified, apply across other cells in the neocortex, change with brain development, and whether these preferred combinations change in different species. Understanding the extent to which preferred combinations of expressed genes vary between neurons could therefore provide a foundation for a genetic classification of neurons.

Although these combinatorial rules do not necessarily indicate binary interactions between the ion channels, they raise interesting questions regarding the transcriptional processes that regulate the expression of the related ion channel genes. For example, they may suggest common or different transcription factors, gene expression promoters and suppressors. However, given that single cell multiplex RT-PCR experiments might introduce a significant number of false negatives, it is very likely that many of the expression rules are not detected. For instance, *Caβ4* and *Caα1A* do not appear to be related in our dataset, although they were previously found to interact [24], [25]. The method is therefore limited in identifying all possible combinatorial expression rules by the quality of the gene expression data. As techniques from single cell transcriptomics improve, it would however become possible to reveal more of the combinatorial rules that govern gene expression. Nonetheless, since false positives are highly unlikely in these experiments we can be confident in the robustness of the combinatorial rules that are identified for the ten neuron types.

The search algorithm is computationally demanding since the size of the search scales quadratically with respect to the number of genes. The algorithm is however highly parallelizable since the models can be computed independently from each other. The algorithm is therefore computationally feasible for analyzing clusters of genes and would require high performance computing to analyze complete transcriptomes in a reasonable time.

In summary, the study provides a novel strategy for the rigorous identification of the expression of preferred combinations of ion channel genes in specific neuronal types, and to reverse engineer their combinatorial expression rules in single neurons. We believe that with additional data the prediction accuracy and power of our iSVM models will greatly improve along with the ability to find additional rules of combinatorial gene expression in other neuron types.

## Materials and Methods

### Single cell data

The dataset used in this analysis was obtained previously in Toledo Rodriguez *et al* [12]. In brief, slices of Wistar rats (13–16 days old) were obtained as described previously in [26], [27]. Somatic whole-cell recordings and histological procedures were performed on 203 neocortical neurons in layers 2 to 6 as described in [26], [27], [28]. Binary genetic profiles of 26 voltage-gated ion channels were obtained using non-quantitative single-cell multiplex RT-PCR. These 203 neurons were selected based on the expression of the house-keeping gene *Gapdh* and a minimum of two ion channel genes. The official Gene Symbols, Names and GenBank Accession No. of the ion channel genes are listed in (Table S5). Each neuron was classified on the basis of its layer of origin, morphological class and electrical firing type. Morphological and electrical type classifications were done as described in [13], [14].

### Data pre-processing

The neurons belonged to 4 different electrical phenotypic profiles (E), 4 different morphological structures (M) and in 4 layers. Neurons were excluded from the analysis if there was no information regarding all three defining characteristics; layer, morphology and electrical firing types. This reduced the sample size from 203 to 135. Neurons were then grouped based on the combination of layer (L), morphology (M), and electrical type (E). This yielded twenty-four types of neuron each with a different number of member neurons. These groups of neurons were then divided depending on the number of neurons (n) per L, M and E combination into a model (n≥4) and a generalization (n<4) datasets (Tables 2 & 3).

Model data consisted of sixty-five neurons belonging to ten different LME combinations each having a minimum of four neurons. The generalization data consisted of eighteen of the remaining neurons that belonged to fourteen different LME combinations with only one or two neurons per combination. This selection was made to ensure that the fourteen neuronal types of the generalization dataset consisted of different combinations of L, M and E used in the model dataset. We did not include LME combinations that have only one or two neurons in the model dataset in order not to induce a bias in our models and also to assess the generalization performance of our models. We also could not use the seventy remaining neurons because their morphological or electrical type was not represented in the model dataset. Given that the L, M and E variables are categorical, we cannot test our predictions on new L, M and E types but only on new and different combinations of L, M and E types in the model dataset. Although this is the largest single cell multiplex study on neurons at this level of characterization, the generalization data contains mostly single examples. Nonetheless, since false positives are highly unlikely in these experiments we expect more under-fitting than over-fitting and hence a larger dataset can only improve the confidence in the results. The results we report therefore provide the most conservative view of the expression rules.

### Model building

We first built a Logistic Regression (LR) and Support Vector Machine (SVM) classifiers for every ion channel gene by using three categorical variables: layer, morphology, and electrical type as input parameters and the binary expression of the ion channel gene as the output parameter. The SVM classifiers were trained and tuned using the radial and linear basis kernels. The optimal cost (*C*) and gamma (*γ*) hyper-parameters were obtained for every model after evaluating 961 grid points (31×31) over the range [2e-15, 2e15] for each of the parameters. Given the low expression frequencies of the ion channel genes, we chose the average expression frequency (0.23) (see *Results*) as the cutoff in the LR classification. The best classifier was selected based on the average 10-fold cross validation accuracy and it came out to be the SVM model. In order to capture the expression diversity within a given LME type and to improve the prediction accuracy we checked whether the expression of a given ion channel gene is affected or not by that of another gene. For that we incremented the number of input parameters by combining the expression of every gene to the three input parameters (L, M, E) and estimated the prediction accuracy using 10-fold cross validation. If the accuracy is improved we retain the combined gene otherwise we reject it. We iteratively combine more genes until the prediction accuracy is no longer improved (Figure 3). This model is referred to as iSVM (incremental Support Vector Machine) model.

### Model assessment

In order to assess the performance of the iSVM models we incremented the basic SVM models with random input genes and used random hyper-parameters and recomputed the prediction accuracy, this model is referred to as riiSVM. We also randomly generated binary data for each gene with a Bernoulli distribution having a probability of expression equal to the observed experimental expression frequency in order to check how the iSVM performance compares to a random data model. The random data was also divided into modeling and generalization data each having the same neuron types and neuron counts as the experimental data, and the random modeling data was used to fit the rdiSVM models. We used analysis of variance (ANOVA) to compare the 10-fold cross validation accuracies of the three models: iSVM, riiSVM, and rdiSVM after 1000 iterations. The accuracies of the iSVM models were also computed for a generalization data set where new combinations of LME parameters not found in the model data are used in order to check whether the identified rules can be generalized or not.

### Network diagram

The indegree of a given ion channel gene is defined to be the number of input genes used in its iSVM model, and the input list is the list of those input genes. The outdegree of a given ion channel gene is defined to be the number of times this gene is used in the iSVM models of all other genes, and the output list is the list of genes that are affected by it. Cytoscape [29] is used to graphically represent the input/output lists of all genes in a directed network diagram.

### Statistical analysis

All statistical tests and models building were done in R 2.11.1 [30]. Student's t-test was used to compute all p-values unless stated otherwise. The packages and functions used in R are listed in Methods S1.

## Supporting Information

Figure S1 **Tuned iSVM parameters and average accuracy of kernels.** ***A*** Average accuracy of the radial and linear kernels for the twenty-six ion channel genes. The radial kernel has a marginally better average accuracy than the linear kernel. ***B*** Distribution of the best gamma parameters identified for the twenty-six channels after tuning the iSVM models over the range [2e-15, 2e15]. ***C*** Distribution of the best cost parameters identified for the twenty-six channels after tuning the iSVM models over the range [2e-15, 2e15].
(TIF)

Figure S2 **Improvement of the average accuracy of the iSVM models after the addition of new input ion channel gene.** The green, blue and black lines correspond to the average accuracy of the SVM, logistic regression (LR) model and a random data iSVM model respectively. The red line represents the mean and standard deviation after 1000 cross validation iterations. Using more than 5 input channels resulted in either a drop or no change in accuracy.
(TIF)

Figure S3 **Heatmap of the Pearson correlation coefficients for the twenty-six ion channel genes.** The maximum absolute coefficient is 0.48.
(TIF)

Figure S4 In situ hybridization stains of *HCN1* (A) and *Kvβ1* (B) from the Allen Brain Atlas P14 mouse developing brain (http://developingmouse.brain-map.org/). The *HCN1* slice is the P14-sagittal-115 slice and the *Kvβ1* slice is the P14-sagittal-104 (Kcnab1) slice. The expression patterns in the somatosensory neocortical area are similar and consistent with the identified rule *HCN1* = *Kvβ1* in our model.
(TIF)

Figure S5 In situ hybridization stains of *Kv1.2* (A), *Kv2.2* (B) and *Kv3.2* (C) from the Allen Brain Atlas P14 mouse developing brain (http://developingmouse.brain-map.org/). The *Kv1.2* slice is the P14-sagittal-137 (Kcna2) slice, the *Kv2.2* slice is the P14-sagittal-142 (Kcnb2) slice and the *Kv3.2* slice is the P14-sagittal-127 (Kcnc2) slice. The three genes have relatively similar expression patterns in the somatosensory neocortical area which is partly in line with our extracted rule (*Kv1.2* = *Kv2.2* AND *Kv3.1* AND *Kv3.2*). We could not check the expression pattern of *Kv3.1* since no P14 slice was found for it.
(TIF)

Table S1 **Identified patterns of expression rules for the twenty ion channel genes.**
(DOC)

Table S2 **Identified expression rules in the ten neuronal types.**
(DOC)

Table S3 **Expression of** ***Kv1.4*** **and** ***HCN3*** **in two different neuronal subtypes.**
(DOC)

Table S4 **Expression of** ***Caα1A*** **and** ***Kv1.6*** **in 6 PC-cAD neurons.**
(DOC)

Table S5 **Official Gene Symbols, Names and GenBank Accession No. of the ion channels genes used.**
(DOC)

Methods S1 (DOC)

## References

[1] Lai HC, Jan LY (2006). The distribution and targeting of neuronal voltage-gated ion channels.. Nature Reviews Neuroscience.

[2] Trimmer JS, Rhodes KJ (2004). Localization of voltage-gated ion channels in mammalian brain.. Annu Rev Physiol.

[3] Kole MH, Hallermann S, Stuart GJ (2006). Single Ih channels in pyramidal neuron dendrites: properties, distribution, and impact on action potential output.. J Neurosci.

[4] Korngreen A, Sakmann B (2000). Voltage-gated K+ channels in layer 5 neocortical pyramidal neurones from young rats: subtypes and gradients.. J Physiol.

[5] Magee JC, Carruth M (1999). Dendritic voltage-gated ion channels regulate the action potential firing mode of hippocampal CA1 pyramidal neurons.. J Neurophysiol.

[6] Inda MC, DeFelipe J, Munoz A (2006). Voltage-gated ion channels in the axon initial segment of human cortical pyramidal cells and their relationship with chandelier cells.. Proc Natl Acad Sci U S A.

[7] Arlotta P, Molyneaux BJ, Chen J, Inoue J, Kominami R (2005). Neuronal subtype-specific genes that control corticospinal motor neuron development in vivo.. Neuron.

[8] Franz O, Liss B, Neu A, Roeper J (2000). Single-cell mRNA expression of HCN1 correlates with a fast gating phenotype of hyperpolarization-activated cyclic nucleotide-gated ion channels (Ih) in central neurons.. Eur J Neurosci.

[9] Subkhankulova T, Yano K, Robinson HP, Livesey FJ (2010). Grouping and classifying electrophysiologically-defined classes of neocortical neurons by single cell, whole-genome expression profiling.. Front Mol Neurosci.

[10] Rossner MJ, Hirrlinger J, Wichert SP, Boehm C, Newrzella D (2006). Global transcriptome analysis of genetically identified neurons in the adult cortex.. J Neurosci.

[11] Sugino K, Hempel CM, Miller MN, Hattox AM, Shapiro P (2006). Molecular taxonomy of major neuronal classes in the adult mouse forebrain.. Nat Neurosci.

[12] Toledo-Rodriguez M, Blumenfeld B, Wu C, Luo J, Attali B (2004). Correlation maps allow neuronal electrical properties to be predicted from single-cell gene expression profiles in rat neocortex.. Cereb Cortex.

[13] Markram H, Toledo-Rodriguez M, Wang Y, Gupta A, Silberberg G (2004). Interneurons of the neocortical inhibitory system.. Nat Rev Neurosci.

[14] Ascoli GA, Alonso-Nanclares L, Anderson SA, Barrionuevo G, Benavides-Piccione R (2008). Petilla terminology: nomenclature of features of GABAergic interneurons of the cerebral cortex.. Nat Rev Neurosci.

[15] Llinas RR (1988). The intrinsic electrophysiological properties of mammalian neurons: insights into central nervous system function.. Science.

[16] Geiger JR, Melcher T, Koh DS, Sakmann B, Seeburg PH (1995). Relative abundance of subunit mRNAs determines gating and Ca2+ permeability of AMPA receptors in principal neurons and interneurons in rat CNS.. Neuron.

[17] McCullagh P, Nelder JA (1989). Generalized Linear Models.

[18] Chang C-C, Lin C-J (2001).

[19] Alpaydin E (2004). Introduction to machine learning.

[20] Science AIfB (2011). Allen Developing Mouse Brain Atlas.

[21] Arkin A, Shen PD, Ross J (1997). A test case of correlation metric construction of a reaction pathway from measurements.. Science.

[22] Arkin A, Ross J (1995). Statistical Construction of Chemical-Reaction Mechanisms from Measured Time-Series.. Journal of Physical Chemistry.

[23] Patrick M, Jagesh S, Robert B, Alberto SV (1993). Espresso-signature: a new exact minimizer for logic functions.. IEEE Transactions on Very Large Scale Integration (VLSI) Systems.

[24] Walker D, Bichet D, Campbell KP, De Waard M (1998). A beta 4 isoform-specific interaction site in the carboxyl-terminal region of the voltage-dependent Ca2+ channel alpha 1A subunit.. J Biol Chem.

[25] Walker D, Bichet D, Geib S, Mori E, Cornet V (1999). A new beta subtype-specific interaction in alpha1A subunit controls P/Q-type Ca2+ channel activation.. J Biol Chem.

[26] Gupta A, Wang Y, Markram H (2000). Organizing principles for a diversity of GABAergic interneurons and synapses in the neocortex.. Science.

[27] Markram H, Lubke J, Frotscher M, Roth A, Sakmann B (1997). Physiology and anatomy of synaptic connections between thick tufted pyramidal neurones in the developing rat neocortex.. The Journal of physiology.

[28] Wang Y, Gupta A, Toledo-Rodriguez M, Wu CZ, Markram H (2002). Anatomical, physiological, molecular and circuit properties of nest basket cells in the developing somatosensory cortex.. Cereb Cortex.

[29] Shannon P, Markiel A, Ozier O, Baliga NS, Wang JT (2003). Cytoscape: a software environment for integrated models of biomolecular interaction networks.. Genome research.

[30] R Development Core Team (2010). R: A Language and Environment for Statistical Computing.

