# Supplementary figures and images for: Combinatorial Expression Rules of Ion Channel Genes in Juvenile Rat (Rattus norvegicus) Neocortical Neurons
Source: PLoS One. 2012 Apr 11;7(4):e34786. doi: 10.1371/journal.pone.0034786 (PMC3324541; doi:10.1371/journal.pone.0034786)

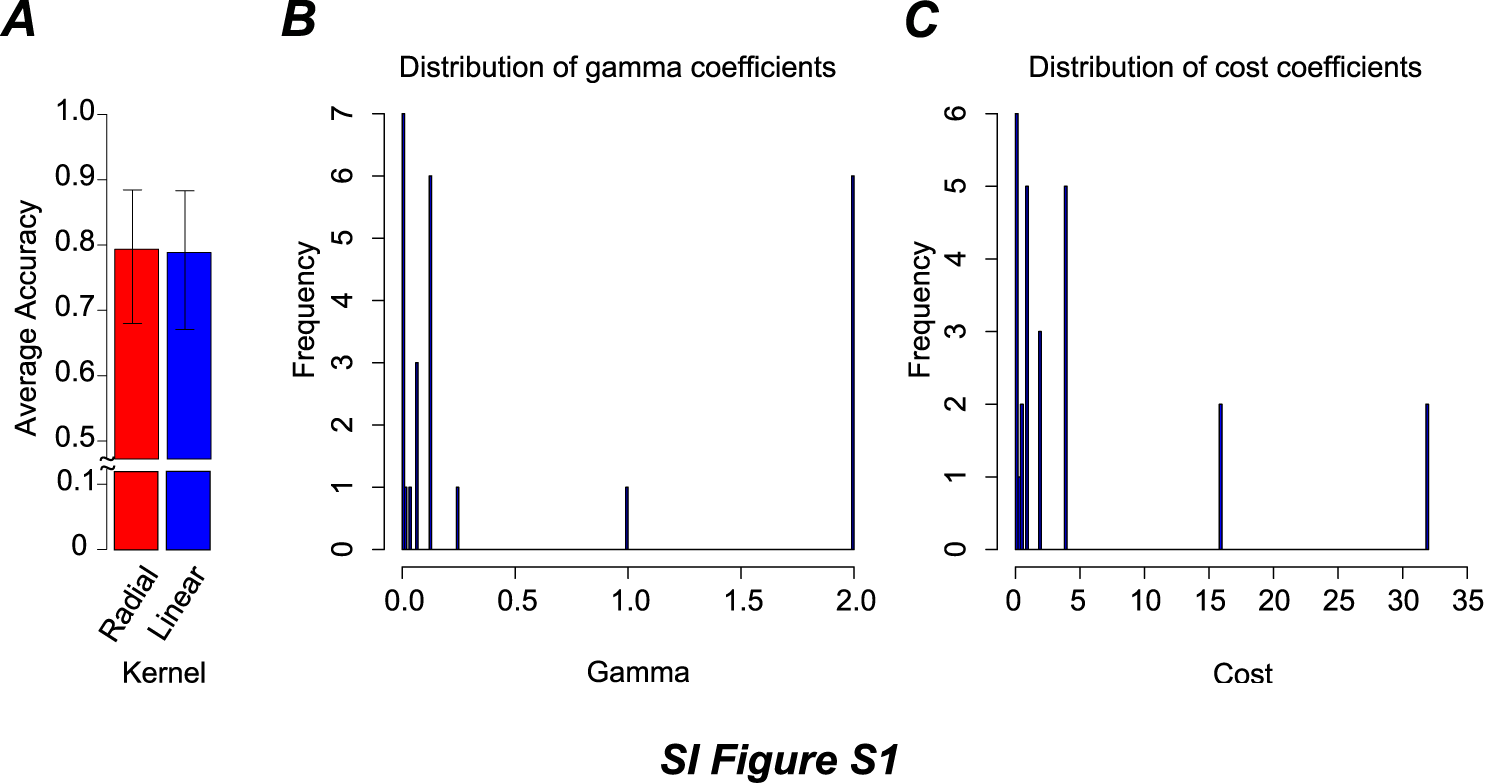

Supplement: Figure S1 — Tuned iSVM parameters and average accuracy of kernels. A Average accuracy of the radial and linear kernels for the twenty-six ion channel genes. The radial kernel has a marginally better average accuracy than the linear kernel. B Distribution of the best gamma parameters identified for the twenty-six channels after tuning the iSVM models over the range [2e-15, 2e15]. C Distribution of the best cost parameters identified for the twenty-six channels after tuning the iSVM models over the range [2e-15, 2e15]. (TIF) [file pone.0034786.s001.tif]

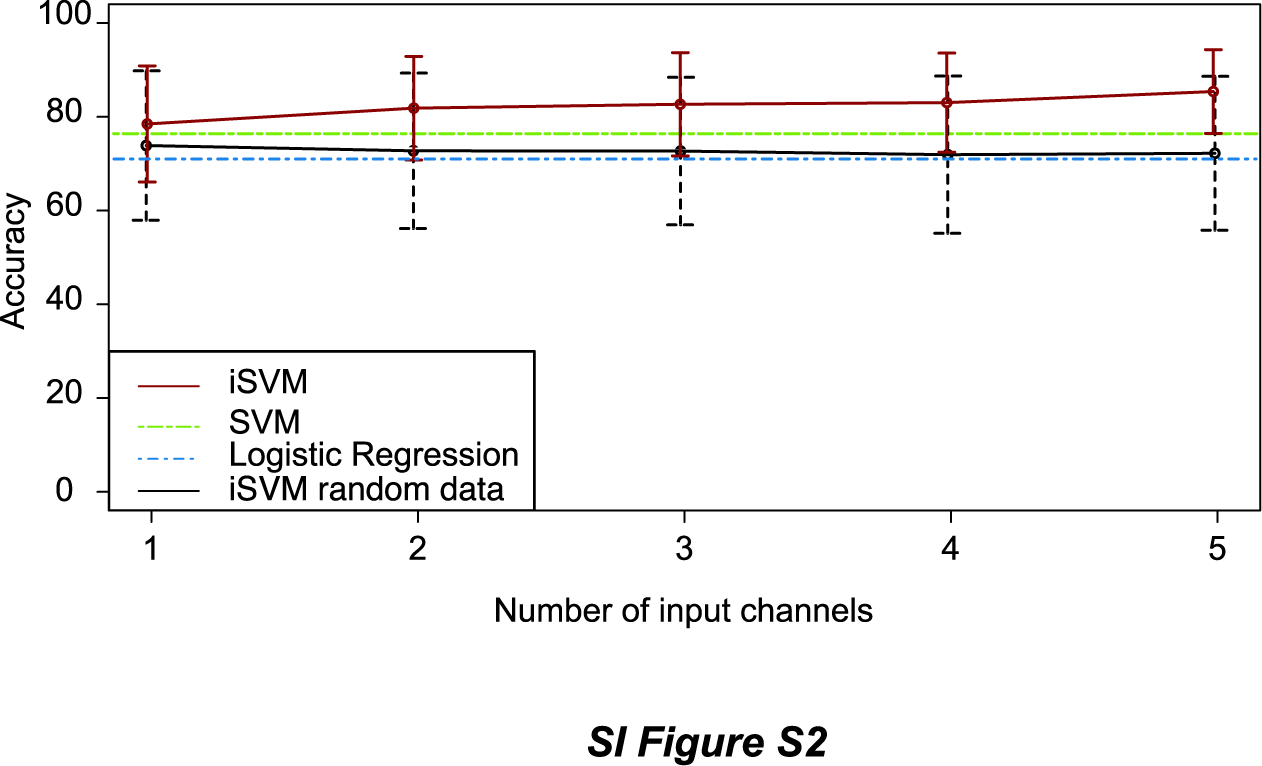

Supplement: Figure S2 — Improvement of the average accuracy of the iSVM models after the addition of new input ion channel gene. The green, blue and black lines correspond to the average accuracy of the SVM, logistic regression (LR) model and a random data iSVM model respectively. The red line represents the mean and standard deviation after 1000 cross validation iterations. Using more than 5 input channels resulted in either a drop or no change in accuracy. (TIF) [file pone.0034786.s002.tif]

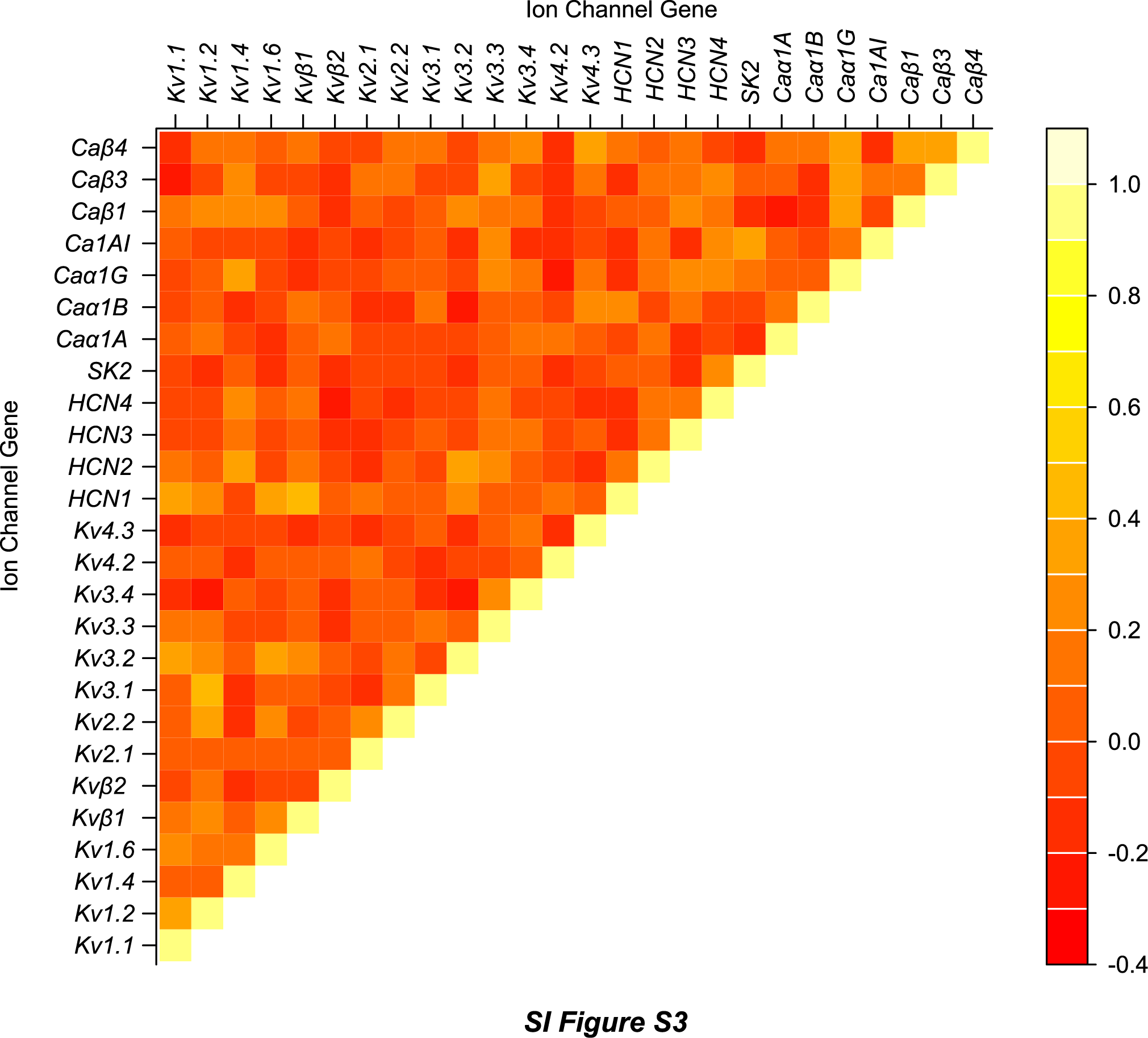

Supplement: Figure S3 — Heatmap of the Pearson correlation coefficients for the twenty-six ion channel genes. The maximum absolute coefficient is 0.48. (TIF) [file pone.0034786.s003.tif]

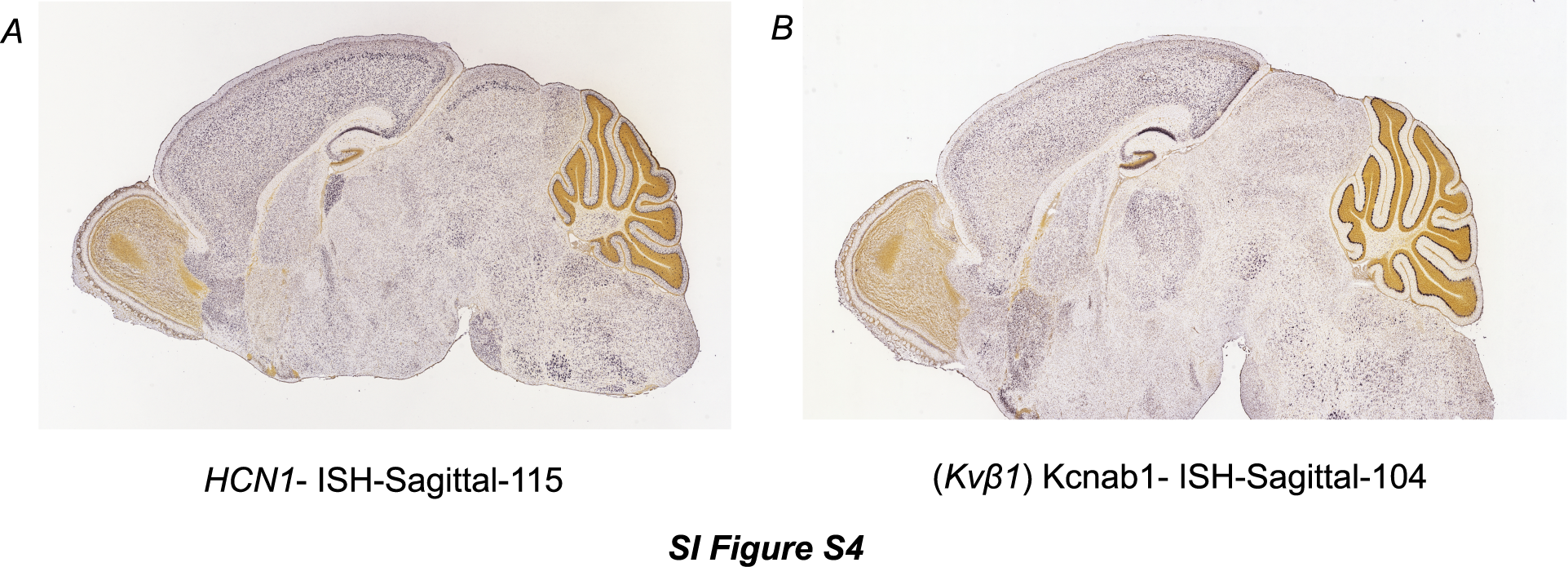

Supplement: Figure S4 — In situ hybridization stains of HCN1 (A) and Kvβ1 (B) from the Allen Brain Atlas P14 mouse developing brain (http://developingmouse.brain-map.org/). The HCN1 slice is the P14-sagittal-115 slice and the Kvβ1 slice is the P14-sagittal-104 (Kcnab1) slice. The expression patterns in the somatosensory neocortical area are similar and consistent with the identified rule HCN1 = Kvβ1 in our model. (TIF) [file pone.0034786.s004.tif]

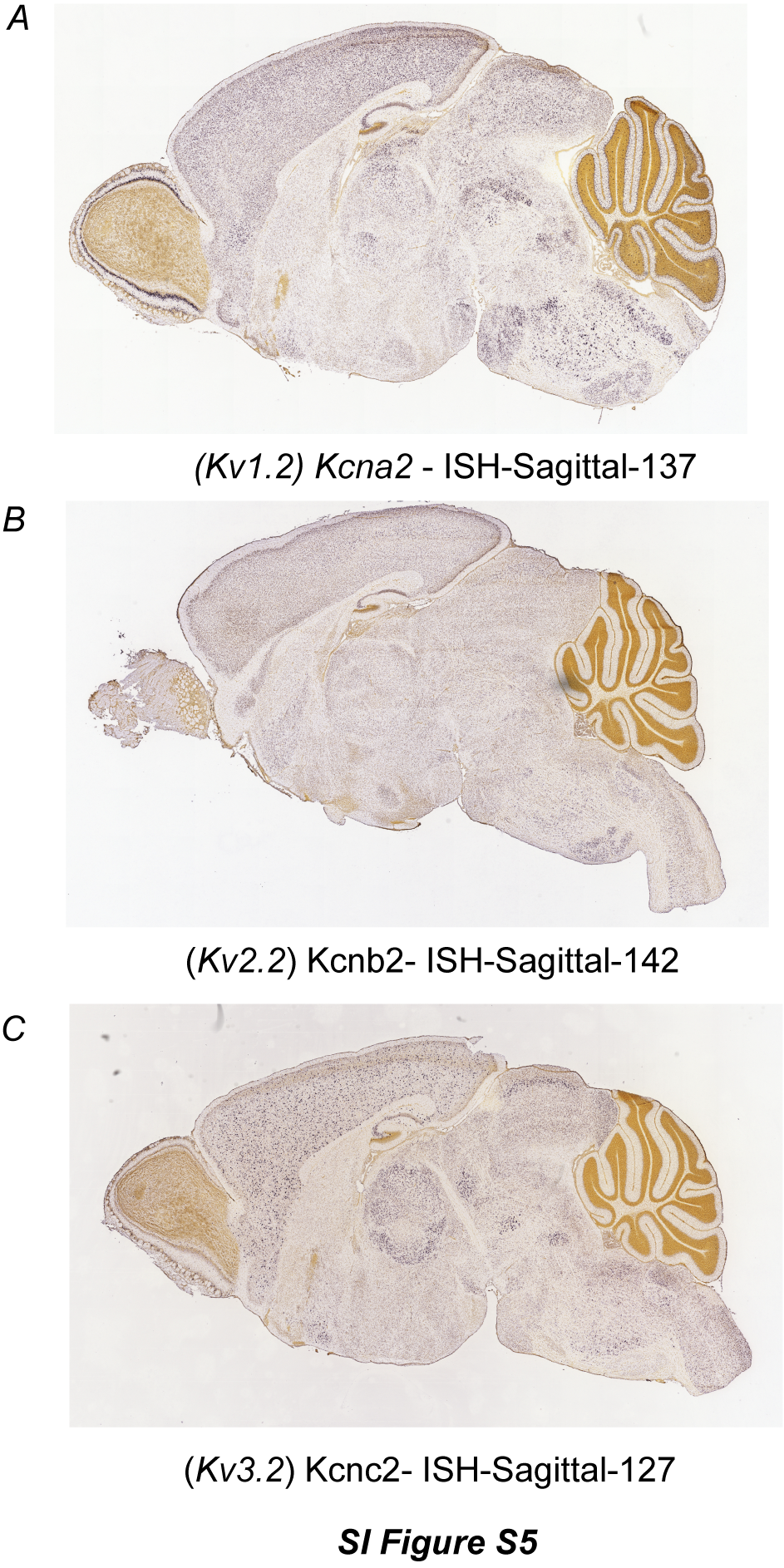

Supplement: Figure S5 — In situ hybridization stains of Kv1.2 (A), Kv2.2 (B) and Kv3.2 (C) from the Allen Brain Atlas P14 mouse developing brain (http://developingmouse.brain-map.org/). The Kv1.2 slice is the P14-sagittal-137 (Kcna2) slice, the Kv2.2 slice is the P14-sagittal-142 (Kcnb2) slice and the Kv3.2 slice is the P14-sagittal-127 (Kcnc2) slice. The three genes have relatively similar expression patterns in the somatosensory neocortical area which is partly in line with our extracted rule (Kv1.2 = Kv2.2 AND Kv3.1 AND Kv3.2). We could not check the expression pattern of Kv3.1 since no P14 slice was found for it. (TIF) [file pone.0034786.s005.tif]
