# Supplementary material for: Combinatorial Expression Rules of Ion Channel Genes in Juvenile Rat (Rattus norvegicus) Neocortical Neurons
Source: PLoS One. 2012 Apr 11;7(4):e34786. doi: 10.1371/journal.pone.0034786 (PMC3324541; doi:10.1371/journal.pone.0034786)
Supplement: Table S1 — Identified patterns of expression rules for the twenty ion channel genes. (DOC) [file pone.0034786.s006.doc]

Table S1. Identified patterns of expression rules for the twenty ion channel genes.

| Ion Channel Gene | Identified Expression Rules | Number of occurrence* |
| --- | --- | --- |
| ***Kv1.4*** | **Kv1.4 = HCN3** in 5MC-cAD and 2/3LBC-dFS neurons. | 8/9 |
| ***Caα1A*** | Caα1A = NOT Kv1.6 in 6 PC-cAD neurons. | 6/6 |
| ***Caβ3*** | **Caβ3 = HCN4** in LBC neurons of layers 2/3 and 4 | 21/22 |
| ***Kv1.1*** | **Kv1.1 = Kv3.2 AND NOT Kv3.4** in 2/3 MC-cAD and 2/3 NBC-cFS neurons. | 17/21 |
| ***Kv2.1*** | **Kv2.1 = NOT Kv2.2 AND NOT Kv3.1** in 6 PC-cAD neurons and 5PC-cAD. | 10/11 |
| ***Kv3.4*** | Kv3.4 = NOT Kvβ2 AND NOT Kv4.2 in 6 PC-cAD neurons.  Kv3.4 = Kv4.2 AND NOT Kvβ2 in 4 MC-cAD neurons. | 6/6  6/6 |
| ***HCN3*** | **HCN3 = Caβ1** in 5 MC-cAD, 2/3 LBC-dFS and 4 MC-cAD neurons.  HCN3 = Caα1B in 2/3 LBC-cAD neurons. | 13/15  6/7 |
| ***Caβ4*** | Caβ4 = Caα1B OR Caβ3 in 6 PC-cAD neurons.  **Caβ4 = Caβ3** in 5 MC-cAD and 2/3 LBC-cFS neurons. | 6/6  8/9 |
| ***Kv1.2*** | **Kv1.2 = Kv3.1 AND Kv3.2** in 5 MC-cAD, 2/3 LBC-dFS, 2/3 LBC-cFS and 4 LBC-cST neurons.  **Kv1.2 = Kv3.1 AND Kv3.2 AND Kv2.2** in 2/3 NBC-cFS, neurons and 2/3 LBC-cAD neurons. | 18/20  19/19 |
| ***Kv1.6*** | **Kv1.6 = Kv3.2 AND (NOT (HCN2) AND NOT (HCN4)** in 2/3 NBC-cFS and 2/3 LBC-cAD neurons. | 16/19 |
| ***Kv4.2*** | Kv4.2=Kvβ1 AND NOT Caβ3 AND NOT Kv3.4 in 6 PC-cAD neurons.  Kv4.2 = Kv3.4 AND NOT Caβ3 AND NOT Kvβ1 in 4 MC-cAD neurons. | 6/6  6/6 |
| ***HCN1*** | **HCN1 = Kvβ1** in 6 PC-cAD, 5 MC-cAD and 5 PC-cAD neurons.  **HCN1= Kvβ1 OR Kv1.1** in 2/3 NBC-cFS and 2/3 LBC-cFS neurons. | 16/16  13/19 |
| ***HCN2*** | HCN2 = NOT Kv3.1 AND NOT Kv2.2 AND NOT Kv4.3 in 5 MC-cAD neurons.  HCN2 = NOT Kv2.2 AND NOT Kv4.3 in 2/3 LBC-cFS neurons.  HCN2 = Kv3.1 AND Kv2.2 AND NOT Kv4.3 in 2/3 LBC-cAD neurons. | 4/5  7/7  7/7 |
| ***Caα1G*** | **Caα1G = Caβ3 AND HCN4** in 4 LBC-cST and 2/3 LB-cFS neurons.  Caα1G = (Caβ3 AND HCN4) OR Caα1B in 2/3 LBC-cAD neurons. | 11/11  7/7 |
| ***Caβ1*** | **Caβ1 = Kv3.2 AND Caβ4 AND NOT Kv2.2** in 5 MC-cAD, 2/3 MC-cAD, 2/3 LBC-dFS, and 2/3 LBC-cFS neurons. | 23/25 |
| ***Kv3.3*** | Kv3.3= (Kv2.2 AND Caβ3) OR KV1.1 in 6 PC-cAD neurons.  Kv3.3 = Kv2.2 OR Kv1.1 in 2/3 MC-cAD neurons.  Kv3.3 = Kv4.3 OR Kv2.2 OR Kv1.1 OR Caβ3 in 2/3 LBC-cAD neurons. | 6/6  8/9  4/7 |
| ***Kvβ1*** | **Kvβ1 = HCN1** in 6 PC-cAD and 5 MC-cAD and 5 PC-cAD neurons.  Kvβ1 = HCN1 AND HCN2 in 2/3 NBC-cFS neurons. | 16/16  10/12 |
| ***Kv3.1*** | **Kv3.1 = Kv3.2 AND Kv1.2** in 5 MC-cAD and 2/3 LBC-dFS neurons.  Kv3.1 = KV3.2 AND NOT HCN4 in 4 LBC-cST neurons. | 9/9  4/4 |
| ***Kv3.2*** | Kv3.2 = NOT Kv1.1 AND NOT Kv4.3 in 5 MC-cAD neurons.  **Kv3.2 = 1** in 4 LBC-cST and 2/3 LBC-cFS neurons.  Kv3.2 = Kv1.1 OR Kv3.4 in 2/3 NBC-cFS neurons. | 5/5  11/11  11/12 |
| ***HCN4*** | HCN4 = Caα1G in 5 MC-cAD neurons.  HCN4 = 0 in 2/3 LBC-dFS neurons.  HCN4 = Caβ3 AND Caα1G in 2/3 LBC-cFS and 2/3 LBC-cAD neurons. | 5/5  4/4  14/14 |

* The number of occurrence corresponds to the number of times the identified rule is observed in the neuron types. It does not correspond to the number of times the expression was 1.
