# Supplementary material for: Combinatorial Expression Rules of Ion Channel Genes in Juvenile Rat (Rattus norvegicus) Neocortical Neurons
Source: PLoS One. 2012 Apr 11;7(4):e34786. doi: 10.1371/journal.pone.0034786 (PMC3324541; doi:10.1371/journal.pone.0034786)
Supplement: Table S2 — Identified expression rules in the ten neuronal types. (DOC) [file pone.0034786.s007.doc]

Table S2. Identified expression rules in the ten neuronal types.

| Neuronal Type | Identified Expression Rules |
| --- | --- |
| 2/3 LBC-cAD | HCN3 = Caα1B |
|  | **Kv1.2 = Kv2.2 AND Kv3.1 AND Kv3.2** |
|  | Kv1.6 = Kv3.2 AND (NOT HCN2 AND NOT HCN4) |
|  | HCN2 = Kv3.1 AND Kv2.2 AND NOT Kv4.3 |
|  | Caα1G = (Caβ3 AND HCN4) OR Caα1B |
|  | Kv3.3 = Kv4.3 OR Kv2.2 OR Kv1.1 OR Caβ3 |
|  | HCN4 = Caβ3 AND Caα1G |
|  | **Caβ3 = HCN4** |
| 2/3 LBC-cFS | Caβ4 = Caβ3 |
|  | **Kv1.2 = Kv3.1 AND Kv3.2** |
|  | HCN1 = Kvβ1 OR Kv1.1 |
|  | HCN2 = NOT Kv2.2 AND NOT Kv4.3 |
|  | Caβ1 = Kv3.2 AND Caβ4 AND NOT Kv2.2 |
|  | **Kv3.2 = 1** |
|  | HCN4 = Caβ3 AND Caα1G |
|  | **Caβ3 = HCN4** |
| 2/3 LBC-dFS | **HCN3 = Caβ1** |
|  | Kv1.2 = Kv3.1 AND Kv3.2 |
|  | Caβ1 = Kv3.2 AND Caβ4 AND NOT Kv2.2 |
|  | Kv3.1 = Kv3.2 AND Kv1.2 |
|  | HCN4 = 0 |
|  | **Kv1.4 = HCN3** |
|  | **Caβ3 = HCN4** |
| 2/3 MC-cAD | Caβ1 = Kv3.2 AND Caβ4 AND NOT Kv2.2 |
|  | Kv3.3 = Kv2.2 OR Kv1.1 |
|  | **Kv1.1 = Kv3.2 AND NOT Kv3.4** |
| 2/3 NBC-cFS | **KV1.2 = Kv2.2 AND Kv3.1 AND Kv3.2** |
|  | Kv1.6 = Kv3.2 AND (NOT (HCN2) AND NOT (HCN4) |
|  | **HCN1 = Kvβ1 OR Kv1.1** |
|  | Kvβ1 = HCN1 AND HCN2 |
|  | Kv3.2 = Kv1.1 OR Kv3.4 |
|  | **Kv1.1 = Kv3.2 AND NOT Kv3.4** |
| 4 LBC-cST | **Kv1.2 = Kv3.1 AND Kv3.2** |
|  | Caα1G = Caβ3 AND HCN4 |
|  | Kv3.1 = KV3.2 AND NOT HCN4 |
|  | **Kv3.2 = 1** |
| 4 MC-cAD | Kv3.4 = Kv4.2AND NOT Kvβ2 |
|  | **HCN3 = Caβ1** |
|  | Kv4.2 = Kv3.4 AND NOT Caβ3 AND NOT Kvβ1 |
|  | **Caβ3 = HCN4** |
| 5 MC-cAD | **HCN3 = Caβ1** |
|  | Caβ4 = Caβ3 |
|  | Kv1.2 = Kv3.1 AND Kv3.2 |
|  | **HCN1 = Kvβ1** |
|  | HCN2 = NOT Kv3.1 AND NOT Kv2.2 AND NOT Kv4.3 |
|  | Caβ1 = Kv3.2 AND Caβ4 AND NOT Kv2.2 |
|  | Kv3.1 = Kv3.2 AND Kv1.2 |
|  | Kv3.2 = NOT Kv1.1 AND NOT Kv4.3 |
|  | **Kv1.4 = HCN3** |
|  | HCN4 = Caα1G |
| 5 PC-cAD | **HCN1 = Kvβ1** |
| 6 PC-cAD | Caα1A=NOT Kv1.6 |
|  | Kv2.1 = NOT Kv2.2 AND NOT Kv3.1 |
|  | Kv3.4 = NOT Kvβ2 AND NOT Kv4.2 |
|  | Caβ4= Caα1B OR Caβ3 |
|  | Kv4.2=Kvβ1AND NOT Caβ3 AND NOT Kv3.4 |
|  | **HCN1 = Kvβ1** |
|  | Kv3.3= (Kv2.2 AND Caβ3) OR KV1.1 |
|  |  |
