# Supplementary material for: Combinatorial Expression Rules of Ion Channel Genes in Juvenile Rat (Rattus norvegicus) Neocortical Neurons
Source: PLoS One. 2012 Apr 11;7(4):e34786. doi: 10.1371/journal.pone.0034786 (PMC3324541; doi:10.1371/journal.pone.0034786)
Supplement: Table S3 — Expression of Kv1.4 and HCN3 in two different neuronal subtypes. (DOC) [file pone.0034786.s008.doc]

Table S3. Expression of *Kv1.4* and *HCN3* in two different neuronal subtypes

| *Kv1.4* SVM | *Kv1.4* iSVM | *Kv1.4* Observed | *HCN3* | Layer | Morphology | Electrical Type |
| --- | --- | --- | --- | --- | --- | --- |
| 0 | 1 | 1 | 1 | 5 | MC | cAD |
| 0 | 0 | 0 | 0 | 5 | MC | cAD |
| 0 | 0 | 0 | 0 | 5 | MC | cAD |
| 0 | 0 | 0 | 0 | 5 | MC | cAD |
| 0 | 1 | 1 | 1 | 5 | MC | cAD |
| 0 | 0 | 1 | 0 | 2/3 | LBC | dFS |
| 0 | 0 | 0 | 0 | 2/3 | LBC | dFS |
| 0 | 0 | 0 | 0 | 2/3 | LBC | dFS |
| 0 | 1 | 1 | 1 | 2/3 | LBC | dFS |

*Kv1.4 is expressed in 5MC-cAD and 2/3LBC-dFS neurons whenever HCN3 is expressed*
