# Supplementary material for: Combinatorial Expression Rules of Ion Channel Genes in Juvenile Rat (Rattus norvegicus) Neocortical Neurons
Source: PLoS One. 2012 Apr 11;7(4):e34786. doi: 10.1371/journal.pone.0034786 (PMC3324541; doi:10.1371/journal.pone.0034786)
Supplement: Table S4 — Expression of Caα1A and Kv1.6 in 6 PC-cAD neurons. (DOC) [file pone.0034786.s009.doc]

Table S4. Expression of *Caα1A* and *Kv1.6* in 6 PC-cAD neurons

| *Caα1A* SVM | *Caα1A* iSVM | *Caα1A* Observed | *Kv1.6* | Layer | Morphology | Electrical Type |
| --- | --- | --- | --- | --- | --- | --- |
| 1 | 1 | 1 | 0 | 6 | PC | cAD |
| 1 | 1 | 1 | 0 | 6 | PC | cAD |
| 1 | 1 | 1 | 0 | 6 | PC | cAD |
| 1 | 0 | 0 | 1 | 6 | PC | cAD |
| 1 | 1 | 1 | 0 | 6 | PC | cAD |
| 1 | 1 | 1 | 0 | 6 | PC | cAD |

*Caα1A and Kv1.6 have the opposite expression profiles in 6 PC-cAD neurons. Caα1A is expressed only when Kv1.6 is not expressed and vice versa*
