# Supplementary material for: Combinatorial Expression Rules of Ion Channel Genes in Juvenile Rat (Rattus norvegicus) Neocortical Neurons
Source: PLoS One. 2012 Apr 11;7(4):e34786. doi: 10.1371/journal.pone.0034786 (PMC3324541; doi:10.1371/journal.pone.0034786)
Supplement: Table S5 — Official Gene Symbols, Names and GenBank Accession No. of the ion channels genes used. (DOC) [file pone.0034786.s010.doc]

Table S5. Official Gene Symbols, Names and GenBank Accession No. of the ion channels genes used.

| **Ion Channel Gene** | **Gene Symbol** | **Gene Name** | **GenBank Accession No.** |
| --- | --- | --- | --- |
| *Kv1.1* | Kcna1 | potassium voltage-gated channel, shaker-related subfamily, member 1 | M26161 |
| *Kv1.2* | Kcna2 | potassium voltage-gated channel, shaker-related subfamily, member 2 | X16003 |
| *Kv1.4* | Kcna4 | potassium voltage-gated channel, shaker-related subfamily, member 4 | X16002 |
| *Kv1.6* | Kcna6 | potassium voltage gated channel, shaker related subfamily, member 6 | X17621 |
| *Kv2.1* | Kcnb1 | potassium voltage gated channel, Shab-related subfamily, member 1 | X16476 |
| *Kv2.2* | Kcnb2 | potassium voltage gated channel, Shab-related subfamily, member 2 | M77482 |
| *Kv3.1* | Kcnc1 | potassium voltage gated channel, Shaw-related subfamily, member 1 | X62840 |
| *Kv3.2* | Kcnc2 | potassium voltage gated channel, Shaw-related subfamily, member 2 | X62839 |
| *Kv3.3* | Kcnc3 | potassium voltage gated channel, Shaw-related subfamily, member 3 | M84211 |
| *Kv3.4* | Kcnc4 | potassium voltage gated channel, Shaw-related subfamily, member 4 | X62841 |
| *Kv4.2* | Kcnd2 | potassium voltage-gated channel, Shal-related subfamily, member 2 | S64320 |
| *Kv4.3* | Kcnd3 | potassium voltage-gated channel, Shal-related subfamily, member 3 | U42975 |
| *Kvβ1* | Kcnab1 | potassium voltage-gated channel, shaker-related subfamily, beta member 1 | X70662 |
| *Kvβ2* | Kcnab2 | potassium voltage-gated channel, shaker-related subfamily, beta member 2 | X76724 |
| *HCN1* | HCN1 | hyperpolarization-activated cyclic nucleotide-gated potassium channel 1 | AF247450 |
| *HCN2* | HCN2 | hyperpolarization activated cyclic nucleotide-gated potassium channel 2 | AF247451 |
| *HCN3* | HCN3 | hyperpolarization-activated cyclic nucleotide-gated potassium channel 3 | AF247452 |
| *HCN4* | HCN4 | hyperpolarization activated cyclic nucleotide-gated potassium channel 4 | AF247453 |
| *SK2* | Kcnn2 | potassium intermediate/small conductance calcium-activated channel, subfamily N, member 2 | U69882 |
| *Caα1A* | Cacna1a | calcium channel, voltage-dependent, P/Q type, alpha 1A subunit | M64373 |
| *Caα1B* | Cacna1b | calcium channel, voltage-dependent, N type, alpha 1B subunit | M92905 |
| *Caα1G* | Cacna1g | calcium channel, voltage-dependent, T type, alpha 1G subunit | AF027984 |
| *Caα1I* | Cacna1i | calcium channel, voltage-dependent, T type, alpha 1I subunit | AF086827 |
| *Caβ1* | Cacnb1 | calcium channel, voltage-dependent, beta 1 subunit | X61394 |
| *Caβ3* | Cacnb3 | calcium channel, voltage-dependent, beta 3 subunit | M88751 |
| *Caβ4* | Cacnb4 | calcium channel, voltage-dependent, beta 4 subunit | L02315 |
