# Supplementary material for: Combinatorial Expression Rules of Ion Channel Genes in Juvenile Rat (Rattus norvegicus) Neocortical Neurons
Source: PLoS One. 2012 Apr 11;7(4):e34786. doi: 10.1371/journal.pone.0034786 (PMC3324541; doi:10.1371/journal.pone.0034786)
Supplement: Methods S1 — (DOC) [file pone.0034786.s011.doc]

**SI Supplementary Methods**

All the analysis was done using the statistical program R 2.11.1. The fitting and tuning of the SVM models was performed using the svm() and tune.svm() functions from the ‘e1071’ package. The *type* parameter used in these functions was “C-classification” and the range of the gamma and cost parameters used for tuning was [2e-15, 2e15]. The 10-fold cross-validation was done by setting the tuning parameter to ‘cross’ with cross=10 (tunecontrol=tune.control(sampling = "cross"), cross=10). Both linear and radial kernels were tested when tuning the SVM models and the radial one was chosen since it was marginally better than the linear one. The logistic regression (LR) model fitting was performed using the glm() function from the ‘stats’ package with the parameter family=“binomial” and the 10-fold cross-validation of the LR models was done using the cv.glm() function from the ‘boot’ package. The accuracy of the SVM, iSVM and LR models was computed as 1-error of the cross-validation. The Receiver Operating Characteristic (ROC) curve and the Area Under the Curve (AUC) were obtained using the ‘ROCR’ package.
